# *In silico* Identification of the Indispensable Quorum Sensing Proteins of Multidrug Resistant *Proteus mirabilis*

**DOI:** 10.3389/fcimb.2018.00269

**Published:** 2018-08-07

**Authors:** Shrikant Pawar, Md. Izhar Ashraf, Shama Mujawar, Rohit Mishra, Chandrajit Lahiri

**Affiliations:** ^1^Department of Computer Science, Georgia State University, Atlanta, GA, United States; ^2^Department of Biology, Georgia State University, Atlanta, GA, United States; ^3^Department of Computer Applications, B.S. Abdur Rahman Crescent Institute of Science and Technology, Chennai, India; ^4^Theoretical Physics, The Institute of Mathematical Sciences, Chennai, India; ^5^Department of Biological Sciences, Sunway University, Petaling Jaya, Malaysia; ^6^Department of Bioinformatics, G.N. Khalsa College, University of Mumbai, Mumbai, India

**Keywords:** *Proteus mirabilis*, urinary tract infection, quorum sensing, eigenvector centrality, k-core analysis

## Abstract

Catheter-associated urinary tract infections (CAUTI) is an alarming hospital based disease with the increase of multidrug resistance (MDR) strains of *Proteus mirabilis*. Cases of long term hospitalized patients with multiple episodes of antibiotic treatments along with urinary tract obstruction and/or undergoing catheterization have been reported to be associated with CAUTI. The cases are complicated due to the opportunist approach of the pathogen having robust swimming and swarming capability. The latter giving rise to biofilms and probably inducible through autoinducers make the scenario quite complex. High prevalence of long-term hospital based CAUTI for patients along with moderate percentage of morbidity, cropping from ignorance about drug usage and failure to cure due to MDR, necessitates an immediate intervention strategy effective enough to combat the deadly disease. Several reports and reviews focus on revealing the important genes and proteins, essential to tackle CAUTI caused by *P. mirabilis*. Despite longitudinal countrywide studies and methodical strategies to circumvent the issues, effective means of unearthing the most indispensable proteins to target for therapeutic uses have been meager. Here, we report a strategic approach for identifying the most indispensable proteins from the genome of *P. mirabilis* strain HI4320, besides comparing the interactomes comprising the autoinducer-2 (AI-2) biosynthetic pathway along with other proteins involved in biofilm formation and responsible for virulence. Essentially, we have adopted a theoretical network model based approach to construct a set of small protein interaction networks (SPINs) along with the whole genome (GPIN) to computationally identify the crucial proteins involved in the phenomenon of quorum sensing (QS) and biofilm formation and thus, could be therapeutically targeted to fight out the MDR threats to antibiotics of *P. mirabilis*. Our approach utilizes the functional modularity coupled with k-core analysis and centrality scores of eigenvector as a measure to address the pressing issues.

## Introduction

Urinary tract infections (UTI) are the second most common infection prevalent amongst long-term hospital patients, second only to pneumonia. Failure to treat or a delay in treatment can result in systemic inflammatory response syndrome (SIRS), which carries a mortality rate of 20–50% (Jacobsen and Shirtliff, 2011; Schaffer and Pearson, 2015)^1^ While *Escherichia coli* remains the most often implicated cause of UTI in previously healthy outpatients, *Proteus mirabilis* take the lead for catheter-associated UTI (CAUTI), causing 10–44% of long-term CAUTIs (Jacobsen and Shirtliff, 2011; Schaffer and Pearson, 2015)^1^. In comparison to normal cases, CAUTI is quite complicated and encountered by patients with multiple prior episodes of UTI, multiple antibiotic treatments, urinary tract obstruction and/or undergoing catheterization as also for those with spinal cord injury or anatomical abnormality (Jacobsen and Shirtliff, 2011; Schaffer and Pearson, 2015)^1^. Such complications of CAUTI caused by *P. mirabilis* arise from the usage of a diverse set of virulence factors by the organism to access and colonize the host urinary tract. These include, but are not limited to, urease and stone formation, fimbriae and other adhesins, iron and zinc acquisition, proteases and toxins and biofilm formation (Schaffer and Pearson, 2015). Despite significant advances made for studying *P. mirabilis* pathogenesis, a meager knowledge of its regulatory mechanism poses an urgent and pressing need to come up with unique health intervention processes for such patients.

In attempts to provide such health interventions, longitudinal, and epidemiological studies on *P. mirabilis* have been reported for extended-spectrum β-lactamase (ESBL) and AmpC β-lactamase (CBL) producers (Luzzaro et al., 2009; Wang J. T. et al., 2014) According to these studies, limited therapeutic options are available for management of such CAUTI which in turn reflects the imminent threats of multi-drug resistance (MDR) *P. mirabilis*. Such MDR phenomenon, exhibited by the gram-negative pathogens like *P. mirabilis*, can be attributed, besides other factors, to the blockade provided by the efflux pumps at the extra-cytoplasmic outer membrane for existing antibiotics entries and remainder drugs expulsion (Eliopoulos et al., 2008; Czerwonka et al., 2016). Besides providing MDR, the cases of CAUTI have been complicated by biofilms formed by the pathogenic *P. mirabilis* (Czerwonka et al., 2016). In fact, different lipopolysaccharide structures of the membrane have been implicated to the adherence of the pathogen on to the surfaces causing CAUTI. Furthermore, along with various other components of the membrane, several cytoplasmic factors interplay among themselves to regulate the cell-density dependent gene regulation. This enables the bacteria for cell-to-cell communication, a phenomenon known as quorum sensing (QS) (Rutherford and Bassler, 2012). Besides other phenotypic traits, QS controls the expression of the virulence factors responsible for pathogenesis of *P. mirabilis* (Stankowska et al., 2012). Again, as per other reports, despite producing two cyclic dipeptides and encoding LuxS-dependent quorum sensing molecule, AI-2, during swarming, *P. mirabilis* has been reported to have no strong evidence of QS (Holden et al., 1999; Schneider et al., 2002; Campbell et al., 2009; Schaffer and Pearson, 2015). However, a highly ordered swarm cycle suggests an existing mechanism for multicellular coordination (Rauprich et al., 1996). Thus, the fact that *P. mirabilis* are engaged in biofilm formation which is managed, albeit in parts, through quorum sensing brings out the complexity of CAUTI. To deal with such complexity, analyses of the proteins involved in such phenomenon, known as the protein interaction networks (PINs), can reveal important information about key role players of the phenomenon (Lahiri et al., 2014; Pan et al., 2015).

The indispensable role players of phenomenon like QS can be determined by analyzing the PIN involving the proteins in the pathway to produce the QS inducer. The essentiality of such small protein interactome (SPIN) can be brought about by an analysis for the most biologically relevant protein to target for inhibiting that phenomenon, also known as quorum quenching. Ideally, a determination of the number of interacting partners of a particular protein identifies its *degree* centrality (DC) which correlates with its essential nature in the biological scenario (Jeong et al., 2001). However, a much deeper understanding of the essential nature of a particular protein comes upon analyzing its interaction with other partners in the global network of all proteins. In this study, we have discussed the relevance of other centrality measures like *Betweenness centrality* (BC), *Closeness centrality* (CC), and *Eigenvector centrality* (EC) (Jeong et al., 2001) parameters for SPIN having the genes and proteins involved in quorum sensing. Again, analyses of a stipulated sets of QS proteins for a valuable knowledge about the most indispensable virulence proteins to render as drug targets for the QS phenomenon could be uninformative. This led us to conduct a deep probing of the whole genome of *P. mirabilis* (WGPM) for a global analysis of the encoding proteins. This comprises the k-core analysis approach of whole genome protein interactome (GPIN) decomposition to a core of highly interacting proteins (Seidman, 1983). Furthermore, to identify the functional modules in the global network (Guimerà and Nunes Amaral, 2005a), we have performed cartographic analyses and predicted the importance of few proteins sharing similar functional modules. To sum up, the sole objective of this study is to utilize several network based models to analyze and identify crucial role players of QS in *P. mirabilis* and thus, propose their importance as potential drug targets.

## Materials and methods

### Dataset collection

The *P. mirabilis* QS pathways for autoinducer-2 (AI-2) biosynthesis were collected from curated reference databases of genomes and metabolic pathways like KEGG, MetaCyc and BioCyc (Caspi et al., 2015; Kanehisa et al., 2015, 2016). The proteins involved in these pathways were extracted with their annotated names and identification as per UniProt database and submitted as entries for the STRING 10.5 biological meta-database (Szklarczyk et al., 2016) to retrieve protein interaction datasets with at least 10 or 50 interactors having the default medium (0.4) level confidence about the interaction, where the interactor numbers relate to the interacting proteins present in the vicinity of the query [period of access: January to February, 2018]. The interactions of the whole genome proteins of the fully annotated *P. mirabilis* strain HI4320 were retrieved from the detailed protein links file under the accession number 529507 in STRING. The sequenced whole genome of the *P. mirabilis* strain HI4320 contains the profile for the same through its full annotation (Pearson et al., 2008). All proteins data, collected and used for interactome construction hereafter, have been reported in Supplementary Data 1.

### Interactome construction

**Figure 1:**
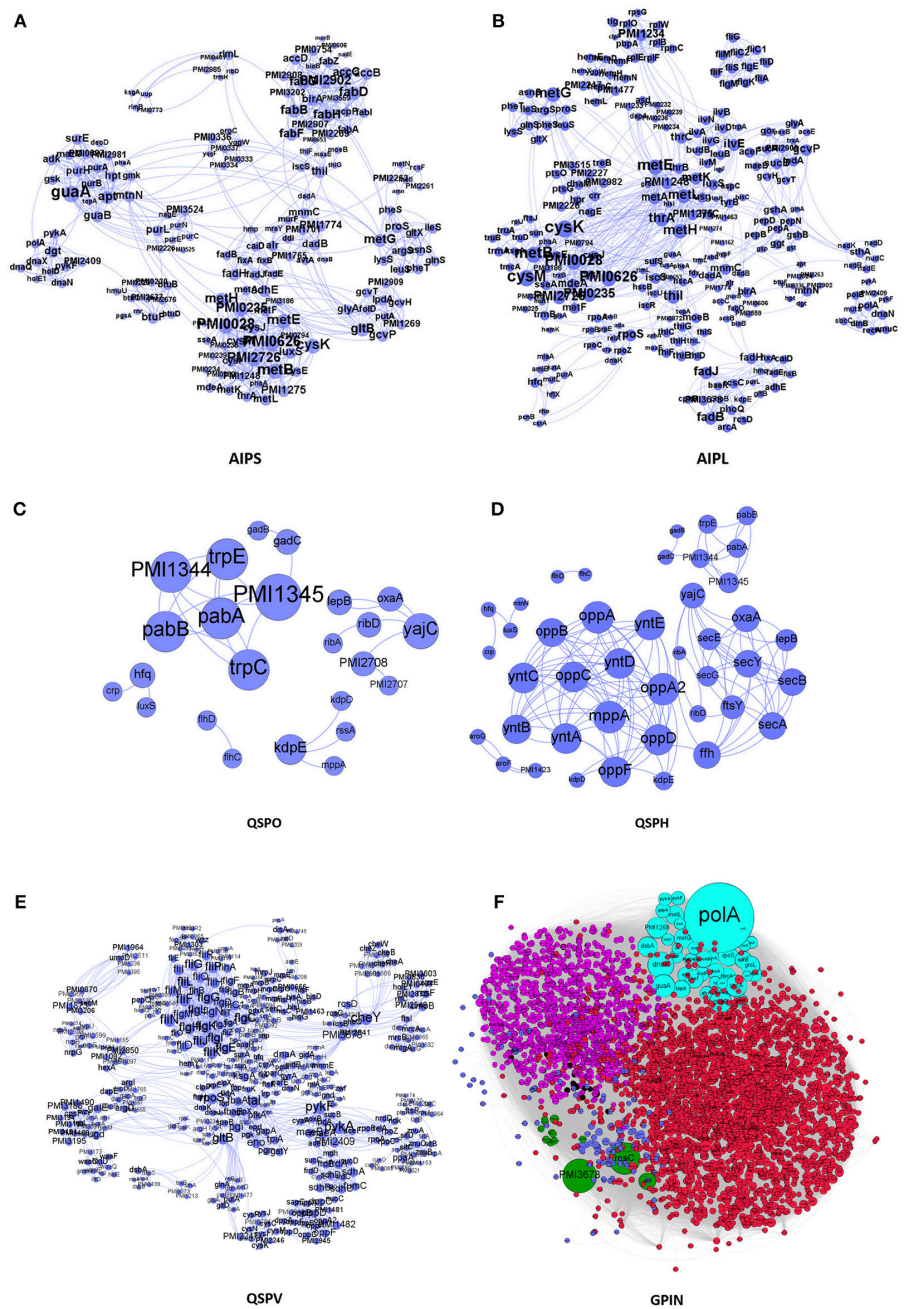
The interactomes of *P. mirabilis* reflecting the degree of connectivity. These comprise the SPIN having proteins coded in light blue colored circles connected to each other through light blue curved lines as in **(A)** AIPS, with 10 interactors from STRING, **(B)** AIPL, with 50 STRING interactors, **(C)** QSPO, having QS genes from *P. mirabilis*, **(D)** QSPH, having *P. mirabilis* homologs reported to be involved in QS of other related species, and **(E)** QSPV, having genes reported to be involved in virulence of *P. mirabilis* (Schaffer and Pearson, 2015). **(F)** GPIN reflecting the 6 different classes (R1–R6) (see Figure 4) of connected proteins in topological space of the network. The six different color codes denote the classes.

We have taken a stepwise approach to integrate and build the interactomes of the proteins, represented by different sections of Figure 1. These are the small protein interactomes (SPIN) comprised of (a) those involved in AI-2 biosynthetic pathway in the organism with small (Holden et al., 1999) and large (Kang et al., 2017) number of interactors retrieved from STRING database (AIPS, AIPL, respectively) (Figures 1A,B), (b) only QS genes found (QSPO) (Figure 1C), (c) all QS genes reported as homologs (QSPH) present in *P. mirabilis* (Figure 1D), (d) all virulence genes reported (QSPV) (Figure 1E) and (e) the WGPM (Figure 1F). Whereas QSPO contains genes reported to be involved in QS in *P. mirabilis*, QSPH contains additional genes reported to be involved in QS in other organisms and present as homologs in *P. mirabilis*. The virulence genes have been taken from the set reported by Schaffer and Pearson (Schaffer and Pearson, 2015). The number of *P. mirabilis* proteins from the SPIN class of interactomes were 31 for AIPS, 42 for AIPL, 24 for QSPO, 42 for QSPH, 58 for QSPV, and 3548 for GPIN (Supplementary Data 1). The medium confidence default values of 0.4 for the individual protein interaction data were obtained from String 10.5. Interactions were 1151 for AIPS, 1571 for AIPL, 30 for QSPO, 129 for QSPH, 2376 for QSPV, and 33462 for GPIN, respectively. These interactions are presented in separate sheets of Supplementary Data 2.

All individual interaction data obtained above were imported into Cytoscape version 3.6.0 (Cline et al., 2007) and Gephi 0.9.2 (Bastian et al., 2009) to integrate, build and analyze five SPIN namely AIPS, AIPL, QSPO, QSPH and QSPV and the GPIN (Figure 1). Interactomes were considered as undirected graphs represented by *G* = (*V, E*) comprising a finite set of *V* vertices and *E* edges where an edge *e* = *(u,v)* connects two vertices *u* and *v* (nodes). In the biological PIN context, a vertex/node represents a protein. The number of physical and functional interactions a protein has with other proteins comprises its degree *d (v)* (Diestel, 2000).

### Network analyses

#### SPIN

**Figure 2:**
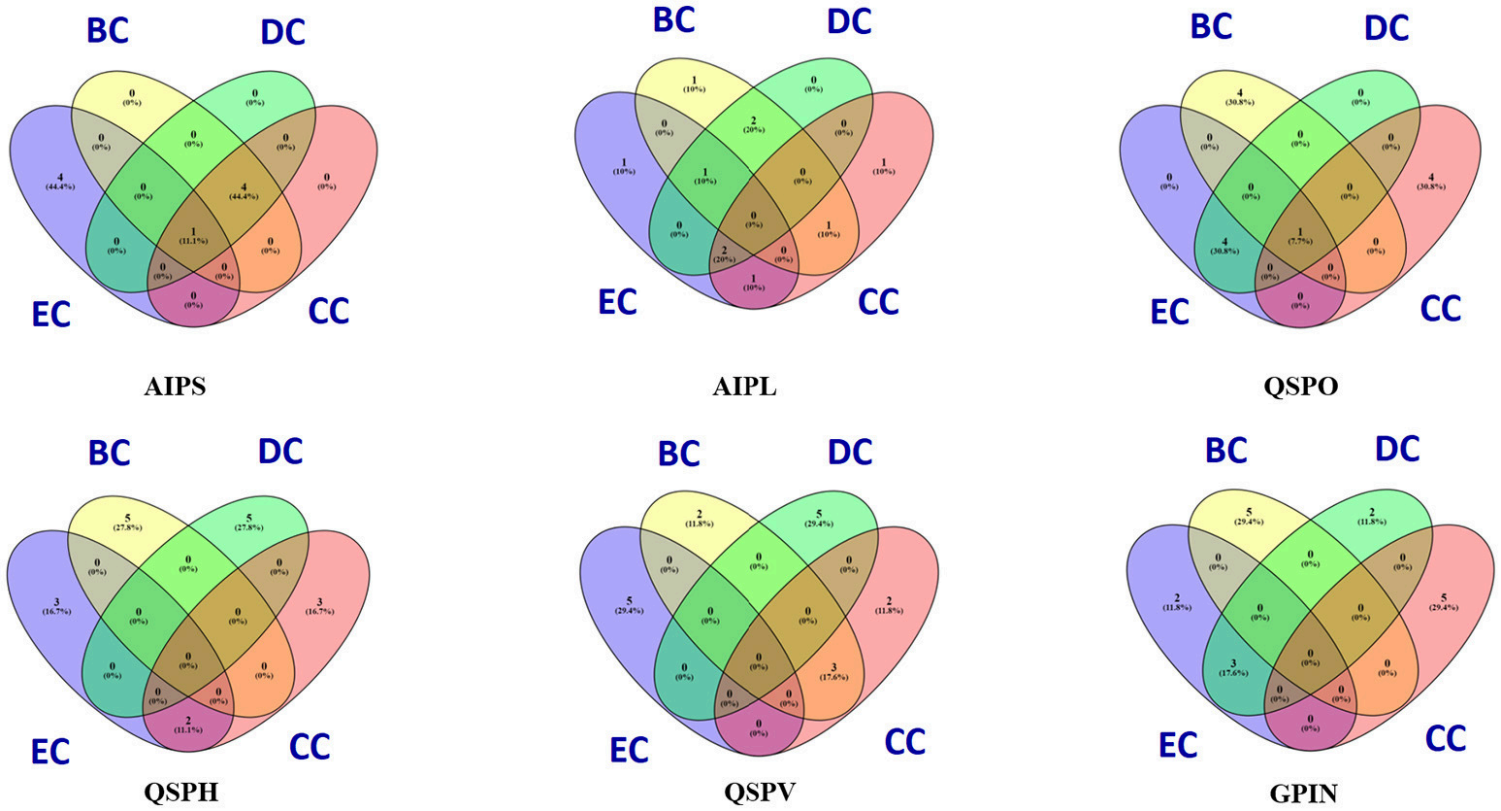
Venn diagram representation for the top five top rankers of BC, CC, DC, and EC parametric analyses of five individual SPIN and GPIN of *P. mirabilis*. BC, CC, DC, and EC stands for betweenness centrality, closeness centrality, degree centrality and eigenvector centrality, degree centrality and closeness centrality, respectively.

The constructed five SPIN were subsequently analyzed individually through the common four measures of centrality applied to biological networks, namely, eigenvector centrality (EC), betweenness centrality (BC), degree centrality (DC) and closeness centrality (CC) (Koschützki and Schreiber, 2004; Özgür et al., 2008; Pavlopoulos et al., 2011; Supplementary Data 3). This was done either via Gephi or the Cytoscape integrated java plugin CytoNCA (Tang et al., 2015). For computing CytoNCA scores, the combined scores obtained from different parameters in STRING were taken as edge weights. The combined scores ranging from 0 to 1, considered in STRING for reporting interactions, generally indicate the confidence of the interaction among the proteins with the level of evidence from the parameters like gene neighborhood, gene fusion, gene co-occurrence, gene co-expression, experiments, annotated pathways and text mining. To find common proteins from each centrality measures, the top 5 proteins were taken for drawing Venn diagrams through online tool Venny 2.1 (Oliveros, 2007–2015) to (Figure 2).

#### GPIN

**Figure 3:**
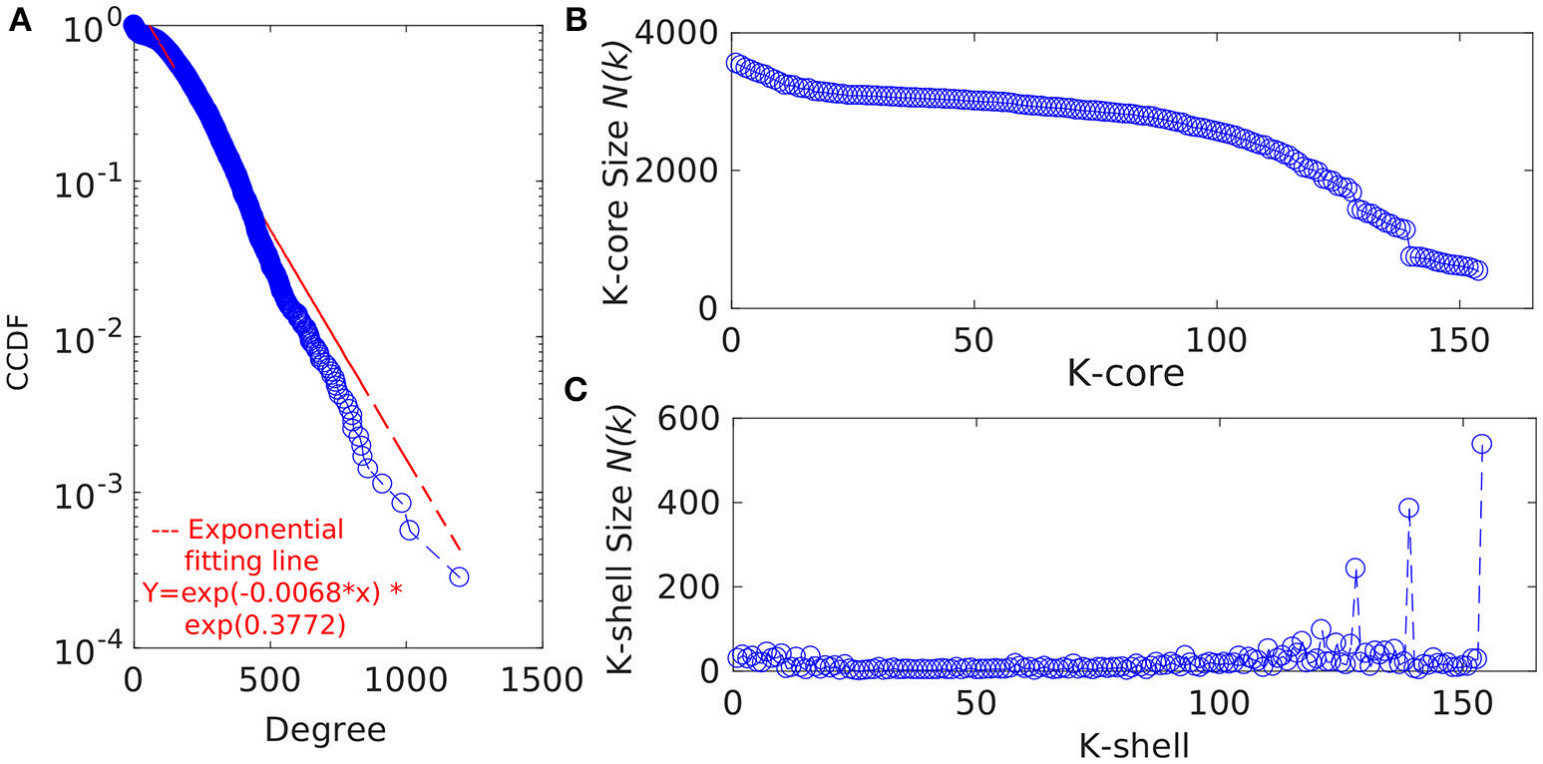
**(A)** The degree distribution of the proteins from the GPIN of *P. mirabilis*. CCDF stands for Complementary Cumulative Distribution Function. Distribution of the **(B)** *k*-core and **(C)** K-shell sizes for the set of proteins from the GPIN of *P. mirabilis*.

**Figure 4:**
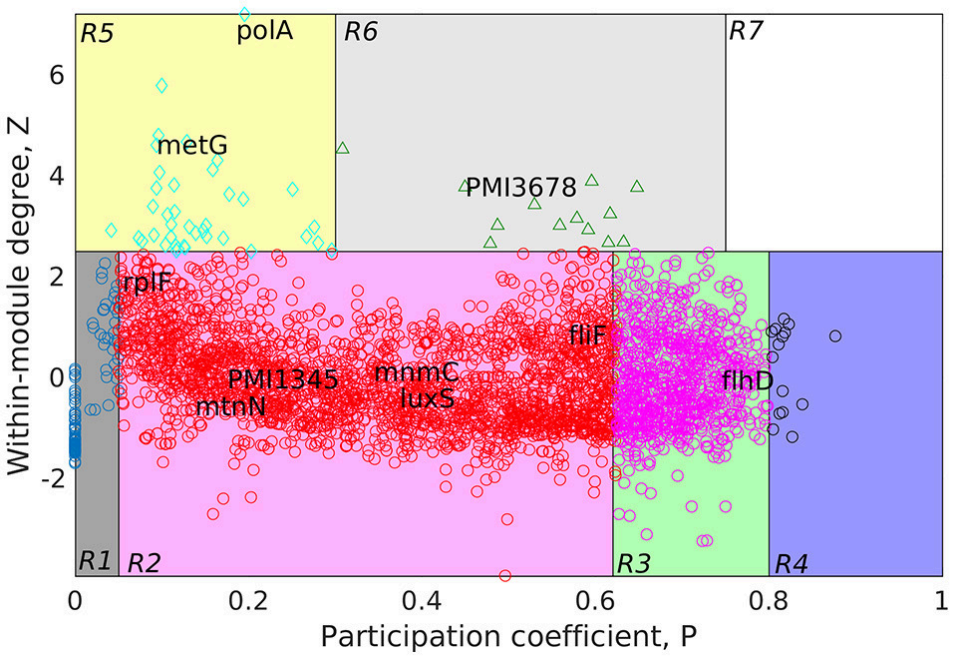
Cartographic representation for classification of proteins from the GPIN of *P. mirabilis* based on its role and region in network space. Quadrants are designated as R1 till R7 with the nodes in each representing different classes of proteins. Colors of quadrants, however, have no significance. Selected topmost proteins, with relevance in QS, biofilm and virulence, analyzed from SPIN are mapped onto different quadrants, as deemed fit as per GPIN analysis.

MATLAB version 7.11, a programming language developed by MathWorks (MATLAB Statistics Toolbox Release, 2010), was used for further analyses of the GPIN. To gain an overview of the technical aspects of the GPIN, the distributions of network degree (k) was plotted against the Complementary Cumulative Distribution Function (CCDF) (Figure 3A). Further concepts about the core group, comprising very specific proteins, was obtained from a *k*-core analysis of the proteins in the whole genome context. This essentially prunes the network to a *k*-core with proteins having degree at least equal to *k* and classifying in K-shell based on their classes of interacting partners (Figures 3B,C). A network decomposition (pruning) technique was adopted to produce gradually increasing cohesive sequence of subgraphs (Seidman, 1983). Further, a significant knowledge of the functional connectivity and participation of each protein was obtained from the network topological representation of the within-module degree z-score of the protein vs. its participation coefficient, P, cartographically represented first by Guimerà and Nunes Amaral (2005b) (Figure 4). The intra-connectivity of a node “i” to other nodes in the same module is measured by the z-score while the positioning of the node “i” in its own module with respect to other modules measures the participation coefficient, P. Participation of each protein reflected its intra- and inter-modular positioning, where functional modules were calculated based on Rosvall method (Rosvall and Bergstrom, 2011). A modular network has high intra-connectivity and sparse inter-connectivity due to which each module has relatively high density and high separability. Each group of nodes in these type of networks share a common biological function as mentioned by Vella et al. (2018). This analysis divided the proteins into mainly two major categories namely the non-hub nodes and hub nodes, where the latter is the connecting point of many nodes. The category of the former has been assigned the roles of ultra-peripheral nodes (R1), peripheral nodes (R2), non-hub connector nodes (R3), and the non-hub kinless nodes (R4). Likewise, the hub nodes have been designated as provincial hubs (R5), connector hubs (R6), and kinless hubs (R7) (Guimerà and Nunes Amaral, 2005b) (Figure 4, Supplementary Data 4).

## Results

To have an understanding of the important protein(s) of QS in *P. mirabilis*, we have taken a stepwise approach of building five SPIN, with an ultimate goal to identify the key role playing proteins in the phenomenon of QS to serve as potential candidates for therapeutic targets. Table 1 represents the comparative picture of the most common topmost proteins, as per centrality measures, in their descending order. In most of the cases, at least three or two of the centrality measures brought out the same protein. These proteins are the ones reflected to be important through each SPIN analysis. For instance, AIPS has MetG and MtnN as the top rankers while LuxS, MnmC, and PMI3678 turns out to be important for AIPL (Table 1). Others like QSPO, QSPH, and QSPV have YajC, PMI1345, OppA, RpoS, flagellar proteins of the *flh and fli* operon and some other two-component systems proteins like CheY and KdpE as important rankers. The functions of these proteins are mentioned in Table 2. The top ranking proteins for each of these five SPINs have been reflected in Figure 2 with Venn diagrams. The common topmost rankers across all the five SPINs are reflected in Supplementary Figure 1.

**Table 1:** The most common topmost proteins of *P. mirabilis* SPIN and GPIN.

**Network**	**EC**	**BC**	**DC**	**CC**
AIPS	**MetG**, LuxS, GcvP, **Hpt**, PMI3524	MtnN, **CysK**, LuxS, MetB, MnmC	MtnN, MnmC, **CysK**, LuxS, MetB	MtnN, **CysK**, LuxS, MetB, MnmC
AIPL	LuxS, **ThrA**, MetH, **MetL**, PMI0028	MnmC, MtnN, LuxS, **PMI3678**, **TrmA**	MnmC, LuxS, MtnN, **ThrA**, MetH	**PMI3678**, **ThrA**, MetH, PMI0028, PMI0626
QSPO	PMI1345, PMI1344, **TrpE**, PabA, **PabB**	YajC, PMI1345, GadC, **RibD**, PMI2708	PMI1345, PMI1344, **TrpE**, PabA, **PabB**	KdpE, **Hfq**, FlhD, FlhC, PMI1345
QSPH	PMI1345, GadC, **TrpE**, FlhD, FlhC	KdpE, **Ffh**, KdpD, **LepB**, **FtsY**	OppA, MppA, OppA2, OppD, OppC	FlhD, FlhC, PMI1423, AroF, AroG
QSPV	FliF, FliK, FlgG, FlgC, FlgI	**RpoS**, **Eno**, Irp, Pgm, **PMI3678**	CheY, PykA, **PykF**, **Tal**, FliN	**RpoS**, **Eno**, **PMI3678**, FliC, CsrA
GPIN	**PolA**, **GuaA**, **DnaK**, **MetG**, **RecA**	**RplP**, PMI1727, PMI1033, PMI2007, **RpoS**	**PolA, PMI3678**, **RcsC**, **DnaK**, **GuaA**	PMI2375, PMI2723, PMI0739, PMI3495, PMI2629

*The bold cased proteins are present in the innermost 154th k- core. EC, BC, DC, and CC stands for eigenvector centrality, betweenness centrality, degree centrality, and closeness centrality, respectively*.

**Table 2:** Functions of centrality based topmost proteins of individual *P. mirabilis* networks.

**Protein name**	**Description of function**
MetG	Is required not only for elongation of protein synthesis but also for the initiation of all mRNA translation through initiator tRNA(fMet) aminoacylation
MtnN	Catalyzes the irreversible cleavage of the glycosidic bond in both 5′-methylthioadenosine (MTA) and S-adenosylhomocysteine (SAH/AdoHcy) to adenine and the corresponding thioribose, 5′-methylthioribose and S-ribosylhomocysteine, respectively
LuxS	Involved in the synthesis of autoinducer 2 (AI-2) which is secreted by bacteria and is used to communicate both the cell density and the metabolic potential of the environment. The regulation of gene expression in response to changes in cell density is called quorum sensing. Catalyzes the transformation of S-ribosylhomocysteine (RHC) to homocysteine (HC) and 4,5-dihydroxy-2,3-pentadione (DPD)
MnmC	Catalyzes the last two steps in the biosynthesis of 5-methylaminomethyl-2-thiouridine (mnm_5_s_2_U) at the wobble position (U34) in tRNA. Catalyzes the FAD-dependent demodification of cmnm_5_s_2_U34 to nm_5_s_2_U34, followed by the transfer of a methyl group from S-adenosyl-L-methionine to nm_5_s_2_U34, to form mnm_5_s_2_U34
PMI3678	Catalyzes the Phosphorelay through sensor kinase activity of two-component Regulatory system
PMI1345	Catalyzes the transfer of the phosphoribosyl group of 5-phosphorylribose-1-pyrophosphate (PRPP) to anthranilate to yield N-(5′-phosphoribosyl)-anthranilate (PRA)
FlhD	Functions in complex with FlhC as a master transcriptional regulator that regulates transcription of several flagellar and non-flagellar operons by binding to their promoter region. Activates expression of class 2 flagellar genes, including fliA, which is a flagellum-specific sigma factor that turns on the class 3 genes. Also regulates genes whose products function in a variety of physiological pathways
FliF	Flagellar protein whose M ring may be actively involved in energy transduction
PolA	In addition to polymerase activity, this DNA polymerase exhibits 5′-3′ exonuclease activity
RplP	Binds 23S rRNA and is also seen to make contacts with the A and possibly P site tRNAs

*The functions of the selected proteins are derived from UniProt database*.

An overview of the important proteins, from individual SPIN as well as across all SPIN, is obtained upon such aforementioned analyses. However, to tackle the MDR *P. mirabilis*, in a global perspective for a drug to be effective, the proteins need to be essentially indispensable. Thus, the whole genome proteins interactome (GPIN) of *P. mirabilis* was then analyzed to understand the global scenario.

### The complete GPIN

In an attempt to analyze the type of network being built from the functional and physical interactions empirically found and theoretically predicted among the whole genome proteins retrieved from STRING, we have observed the degree distribution of GPIN to be exponential showing a non-linear preferential attachment nature (Figure 3A; Vázquez, 2003). Hereafter, we have framed an idea of the important proteins from an array of proteins involved in the five individual SPIN, upon performing a k-core analysis for them (Figures 3B,C). Notably, the innermost core was 154th shell and had genes like *thrA, cysK, metG, metL, trpE, rpoS, eno*, etc. which have already been reflected from the four network centrality analyses of the SPINs (Table 1, Supplementary Data 3: Sheet 1–5). Additionally, it is to be noted that top 5 EC and DC measures of the GPIN also had their position in the innermost 154th core, thereby indicating their importance in the global scenario. Other important genes e.g., *luxS, PMI1345* from the k-core analyses were found in the 139th shell. The latter category was found to have direct involvement in QS.

Furthermore, to classify the proteins based on their regional positioning and functional role in the network topological space of *P. mirabilis*, we have analyzed the GPIN represented cartographically (Figure 4, Supplementary Data 4). Essentially, such representation would classify the complete set of proteins in the genome with respect to their connectivity within similar classes of proteins performing similar biological function (functional module) along with their participation with other related and/or non-related functional module (also see materials and methods and discussion section). Noticeably, the R6 quadrant had the top 5 proteins belonging to either the innermost 154th core or almost close to the 139th core containing most of the proteins related to QS (Supplementary Data 4). These are GltB and PMI3678 for the former and PMI3348, PMI0587, and PMI3517 for the latter. Moreover, upon looking deep into EC classification of R6 quadrants, all top 5 proteins, namely PolA, GuaA, DnaK, MetG, and RecA were from the innermost 154th core. Furthermore, analysis after sorting of module followed by R quadrant, k-core followed by either module or EC measures, all revealed the proteins to be mostly belonging to the R6 or R5 categories, besides their 154th or 139th core classification (Supplementary Data 4). It is worthwhile to mention here that a similar sorting analyses of BC with respect to Quadrant and k-core had revealed proteins mostly from R2 or R3, none of them occupying the innermost 154th core, except RplP, and RpoS.

## Discussion

We have started with the proteins involved in *P. mirabilis* AI-2 biosynthesis pathway (Supplementary Figure 2) and derived the AIPS besides AIPL (Figures 1A,B). While the former connects the proteins of the pathway as reported by default in STRING with only 10 interactors, supposedly directly involved in the phenomenon of AI biosynthesis, the latter has been formed upon extending those to 50 interactors per protein query. The idea was to incorporate other related proteins having connectivity to the AI-2 whose analysis might give more insight about QS in *P. mirabilis*. Moreover, it was necessary to have an idea of the robustness of the proteins involved in QS pathways and thus, QSPO was constructed to have an idea of the proteins directly involved in the phenomenon of QS in *P. mirabilis* only (Figure 1C). Again, with the homologous proteins reported to be involved in QS in other species from KEGG database, it was necessary to look into their association with acknowledged QS proteins of *P. mirabilis* (Supplementary Figure 3). Thus, QSPH was constructed to take into consideration of this fact and analyze further (Figure 1D). Furthermore, with multiple genes and proteins reviewed for the virulence of *P. mirabilis* (Schaffer and Pearson, 2015), including those involved for QS phenomenon, it was necessary to have an interactome QSPV constructed to analyse their interactions and involvement (Figure 1E). All these SPIN were constructed to have an understanding of the indispensable proteins responsible for QS in *P. mirabilis*. Finally, a complete whole genome analyses for other plausible indispensable proteins connecting biofilm formation, AI-2 biosynthesis, quorum sensing and even MDR was necessary to have a bird's eye view of the global scenario. This was done with the construction and analyses of the GPIN (Figure 1F).

The five SPIN were then analyzed individually by utilizing the four important centrality measures of DC, CC, BC, and EC. Of these, DC is the most basic, informing the connectivity of any protein in the network. CC might reflect the proximity of a protein in terms of its communication with others to render a functionally virulent phenotype. Being a comparatively better measure in terms of bridging different functionally important groups of virulent proteins, BC might bring out the importance of a protein to be targeted for therapeutic purposes. However, EC might show the most important proteins having their impact on other important proteins in a virulent network and thus, turn out to be indispensable protein to target. We have found a varying range of proteins ranging from the locomotive flagellar proteins of the *flh* and *flg* operon (Claret and Hughes, 2000), LuxS (Schneider et al., 2002) and MtnN directly involved in AI-2 biosynthetic pathway, MetG and MnmC involved in the protein translation machinery along with the proteins PMI1345 (Wang M. C. et al., 2014) and PMI3678 with catalytic activities/domains, chaperone protein, Hfq (Wang M. C. et al., 2014), signal transduction protein, KdpE (Rhoads et al., 1978), and a pre-protein translocase subunit, YajC (Pearson et al., 2008). Among the proteins PMI1345 and PMI3678, as per UniProt database, the former is having an activity as anthranilate phosphoribosyltransferase catalyzing the transfer of the phosphoribosyl group of 5-phosphorylribose-1-pyrophosphate (PRPP) to anthranilate to yield N-(5′-phosphoribosyl)-anthranilate (PRA). Essentially, PMI1345 is involved in the 2nd step of the subpathway synthesizing L-tryptophan from chorismate. Again, PMI3678 has the histidine kinase domain and displays activities of kinase through ATP binding and in-turn regulates transcription via a two-component regulatory system. Thus, as analyzed above, with the different proteins, pertaining to the biofilm formation, flagellar locomotion, translation and signal transduction, a level of complexity of the *P. mirabilis* QS machinery could be perceived.

To gain more insight into the global scenario of the whole genome, we have constructed the GPIN (Figure 1F) and analyzed it through several network topological and centrality parametric measures (Supplementary Datas 3, 4). For this GPIN, we have observed that the connectivity distribution, P(k), of a particular node gets connected to k other nodes, for large values of k. This confirms that the GPIN is indeed a large network and neither a random, Erdos and Renyi type (Erdos and Rényi, 1960) nor a small-world, Watts and Strogatz type (Watts and Strogatz, 1998). Our GPIN is free of a characteristic scale and roughly followed the power-law (Albert et al., 2000) with an exponential decay of the degree distribution (Figure 3A). Initially, we have analyzed the constructed GPIN with *k*-core/K-shell topological parameters (Figures 3B,C). Technically speaking, a k-core is a subnetwork with a minimum number of k-links. A K-shell is a set of nodes having exactly k-links. In another words, K-shell is the part of k-core but not of (k+1)-core. Thus, proteins belonging to the outer shell have lower k value thereby reflecting the limited number of interacting partner proteins. On the contrary, proteins from the inner *k*-core/K-shell are very specific ones having high interaction with each other and are considered to be the most important ones. It has been observed that the inner core member proteins are highly interactive due their robust and central character (Alvarez-Hamelin et al., 2006). In this light, a complete decomposition of the network, achieved by decomposing the core, would reveal the innermost important part of the network. We have found the 154th core as the innermost one for our GPIN having many proteins involved in the biosynthesis of amino acids, including cysteine and methionine, the amino acid precursor of the components of AI-2 biosynthetic pathway. These proteins rank top for most of the EC measures across the other five SPIN as well. Furthermore, the 139th core was on focus due to its nearby proximity to the innermost core and comprising most of the proteins directly involved in QS. Our analyses till this far revealed LuxS and PMI1345 to be the prominent EC proteins in the 139th core of the genome. Interestingly, only PolA and RplP, top rankers of BC measures, made it to the innermost 154th core compared to the other topmost EC proteins in that core. This probably reflects the importance of EC measure to reveal the prominent stakeholders of the machinery responsible for the very survival and probably virulence of the organism. Any effective drug target should, thus, be selected from this core group with high EC rank.

A further delving deep into the functional connectivity of the modules formed in network topological space reinforced our findings this far. The topological orientation of the nodes in space are being represented cartographically where *P*-values have been put in the x-axis and z-score values in the y-axis. In this context, R1 has low *P*-values and low z-scores while R7 has the highest for both of them. Following this representation, the non-hubs and the hubs are classified into the protein groups of R1-4 and R5-7, respectively. Among them, the kinless hubs proteins (R7), having high connection within module (z) as well as between modules (P) scores, becomes important in terms of functionality. Similarly, the ultra-peripheral proteins (R1), with least P and z measures, are the least connected across the network followed by the peripheral proteins (R2). Such proteins can be detached easily and thus, are perceived, not much to affect the whole network when attempted to reach the core upon decomposition. This is nothing but the outermost shells of the k-core measures (refer previous section) which has proteins not grossly affecting the survival of the organism. Likewise, proteins belonging to the non-hub connectors (R3) group might be involved in only a small but fundamental sets of interactions. On the contrary, proteins of the provincial hubs class (R5) have many connections which are within-module. Again, the non-hub kinless proteins (R4) link other proteins which are evenly distributed across all the modules. However, the connector hub proteins (R6) link most of the other modules and are expected to be the most conserved in terms of decomposition as well as evolution. This could be the very set of proteins which the organism would maintain as the essential ones for their very survival. We have observed mostly R5 and R6 classes of proteins occupying the innermost 154th and the QS-involved 139th cores. Furthermore, the EC measures brings out the importance when compared to other measures of centralities.

In order to bring out the biological implication of the cartographic analyses, we now discuss the relevance of the proteins identified as essential in the context of virulence, biofilm formation and QS phenomenon. In this context, it is important to note that, we have observed many of the already known genes and proteins, viz LuxS, FlhDC to be reflected from our *in silico* cartographic analyses as well. For example, with the highest number (17) of fimbrial operons reported in any sequenced bacterial species, four *P. mirabilis* fimbriae, namely, MR/P, UCA, ATF, and PMF have shown prominent roles in biofilm formation (Scavone et al., 2016). The thickness, structure, and the amount of exopolysaccharides produced by some biofilms formed by *P. mirabilis* are influenced by important acylated homoserine lactones (Stankowska et al., 2012). Moreover, some virulence factors are regulated by QS molecules like acylated homoserine lactones (acyl-HSLs) (Henke and Bassler, 2004). Of the two QS types, LuxS is an essential enzyme for AI-2 type which is coded by luxS gene having S-ribosylhomocysteine lyase activity (Schneider et al., 2002). Acetylated homoserine lactone derivatives modifies the expression of virulence factors of *P. mirabilis* strains (Stankowska et al., 2008). The flhDC master operon is a key regulator in swarmer cell differentiation in *P. mirabilis*, it is known to cause an increased viscosity and intracellular signals (Fraser and Hughes, 1999). Furthermore, the extracellular signals can be sensed by two-component regulators such as RcsC–RcsB (Fraser and Hughes, 1999).

Having said the above, we observe that, many such genes and proteins, not reported to have connections with QS and virulence, have also been unearthed from our study. Thus, it is imperative to have an in-depth analysis to bring out the importance of the proteins unearthed through the process. In order to achieve the same, we rely on the fact that the innermost 154th core could harbor the genes/proteins essential for the very survival of the organism. Moreover, our cartographic analysis shows that R6 classes of proteins having high intra- and inter-connectivity, within and between the functional modules might play a crucial role in the maintenance of the organismal structure. This adds up to another level of indispensable nature. Furthermore, the very concept of Eigenvector centrality, which reflects the important proteins' connectivity with other such important proteins in terms of their function, finalize the indispensable factor. This method of utilizing the *k*-core, functional module and centrality measure, like that of Eigenvector, has been used to analyze large networks to reveal the important proteins, albeit, in a complete different scenario (Ashraf et al., 2018). Utilizing this method, referred to as KFC, we found the three topmost indispensable factors for *P. mirabilis* are *gltB, PMI3678*, and *rcsC* (Supplementary Data 5). It is important to note that the glutamate synthase encoding gene *gltB*, has been shown to be involved in a quorum sensing-dependent glutamate metabolism which affects the homeostatic osmolality and outer membrane vesiculation in *Burkholderia glumae* (Kang et al., 2017). Expression level of *gltB* has been shown to affected in *E. coli* by the stationary phase QS signals (Ren et al., 2004). Again, *rcsC* encodes an sensor histidine kinase protein which is known to be involved in swarming migration and capsular polysaccharide synthesis along with *yojN* (Belas et al., 1998; Fraser and Hughes, 1999). The sensor kinase activity for *PMI3678* encoding an aerobic respiration control protein, however, has not been reported earlier for *P. mirabilis*, and thereby could serve as one of the important therapeutic targets. All these proteins are quite different to those reported to be quite important in a recent study to unearth the fitness factors in a single-species and polymicrobial CAUTI setting, performed with a genome wide transposon mutagenesis of *P. mirabilis* (Armbruster et al., 2017). In this study, Armbuster *et al*. has observed the polyamine uptake and biosynthesis to the fitness factor for single species CAUTI while branched chain amino acid (BCAA) synthesis turned out to be important for polymicrobial infection along with *Providencia stuartii* (Armbruster et al., 2017). None of these fitness factors, found to be helpful in colonizing either the catheterized bladder (referred to as *F*actors for *B*ladder *C*olonization, FBC) or the kidney (*F*actors for *K*idney *C*olonization, FKC), were observed in our analysis to be belonging to the R6 quadrant despite some falling within the innermost 154th core (Supplementary Data 5). While the reports by Armbuster et al. is in a live and dynamic setting, ours is, a static and theoretical network analysis. However, given the fact that this theoretical analysis reflects only a few indispensable ones, they might have some relevance in therapeutic intervention strategies to tackle CAUTI caused by MDR *P. mirabilis*.

## Conclusion

This study takes a stepwise approach to identify the crucial role players from different sets of interacting proteins of *P. mirabilis* involved primarily in QS phenomenon. Essentially, this delineates the building of theoretical interactomes comprising the five individual SPIN which are analyzed through network parametric measures to reveal the most important proteins for such phenotype of QS and biofilm formation. All these lead to the identification of LuxS and PMI1345 to be important proteins of this organism. Furthermore, the results are supplemented through a decomposition of the *P. mirabilis* genome interactome, GPIN, followed by analysis of centrality measurements to reach the innermost core of the proteins essential for virulence and survival. Such in-depth analysis of the GPIN revealed other classes of important conserved proteins like GltB, PMI3678, and RcsC having the potential for being the most important ones and thus, indispensable among the set of whole genome proteins of *P. mirabilis*.

## Author contributions

The analyses and the study were conceptualized, planned and designed by CL. Data generated by SP, MA, SM, and RM were analyzed by CL supported by SM and RM with tabulation. Additional scripts for QC were written by RM. Artwork was done by MA, SM, and SP. CL primarily wrote and edited the manuscript aided by inputs from SP, MA, and SM.

### Conflict of interest statement

The authors declare that the research was conducted in the absence of any commercial or financial relationships that could be construed as a potential conflict of interest.

## Footnotes

^1^https://emedicine.medscape.com/article/226434-overview#a6.
