# Supplementary figures and images for: In silico Identification of the Indispensable Quorum Sensing Proteins of Multidrug Resistant Proteus mirabilis
Source: Front Cell Infect Microbiol. 2018 Aug 7;8:269. doi: 10.3389/fcimb.2018.00269 (PMC6090301; doi:10.3389/fcimb.2018.00269)

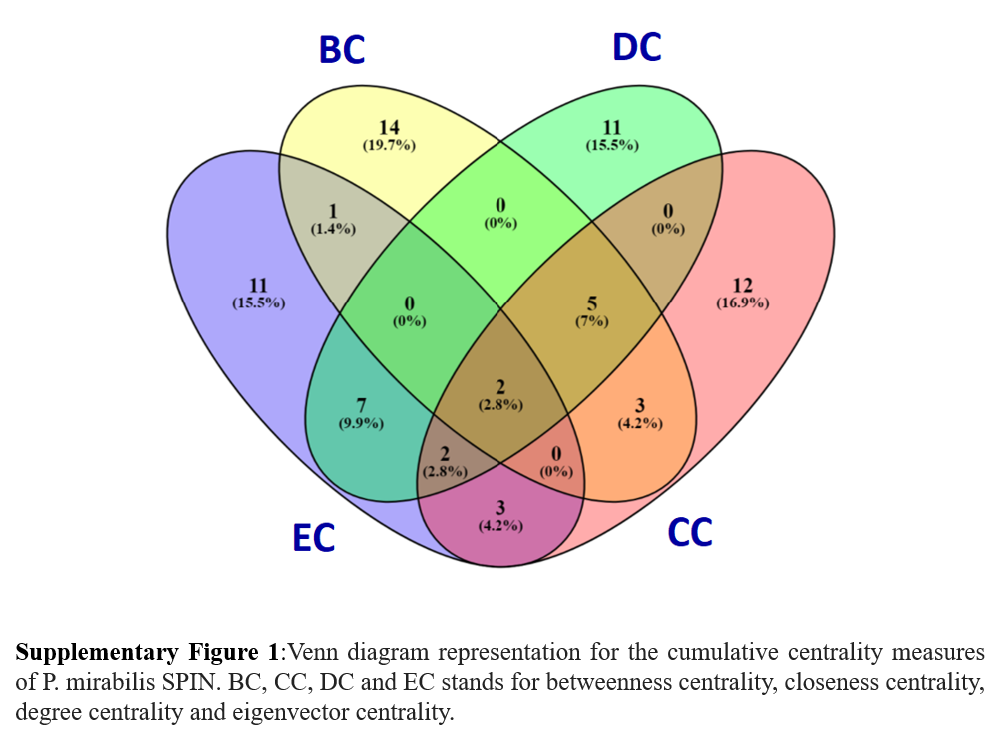

Supplement: Supplementary file 6 [file Image_1.PNG]

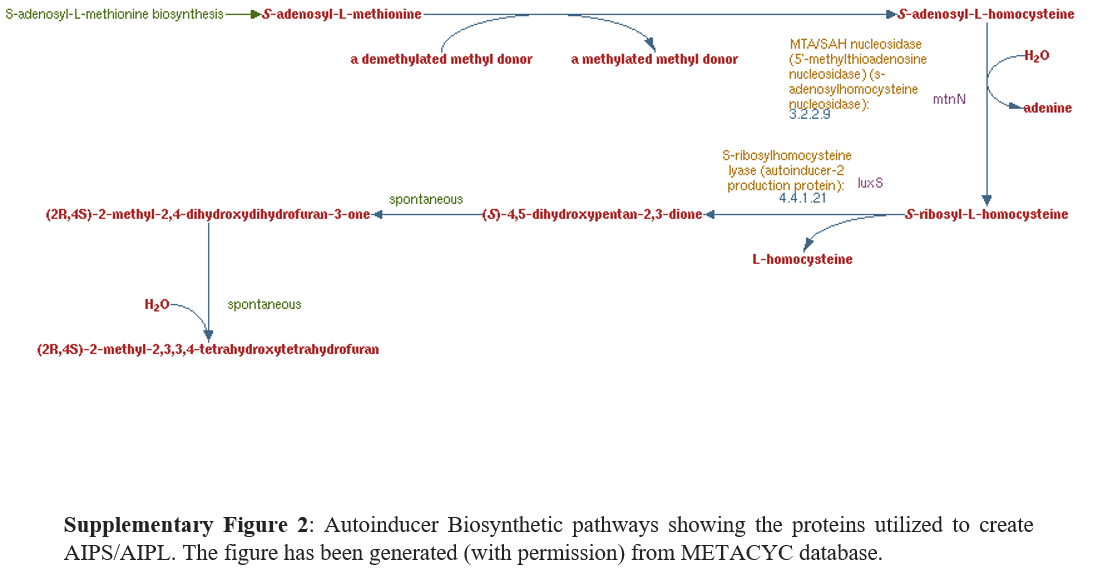

Supplement: Supplementary file 7 [file Image_2.PNG]

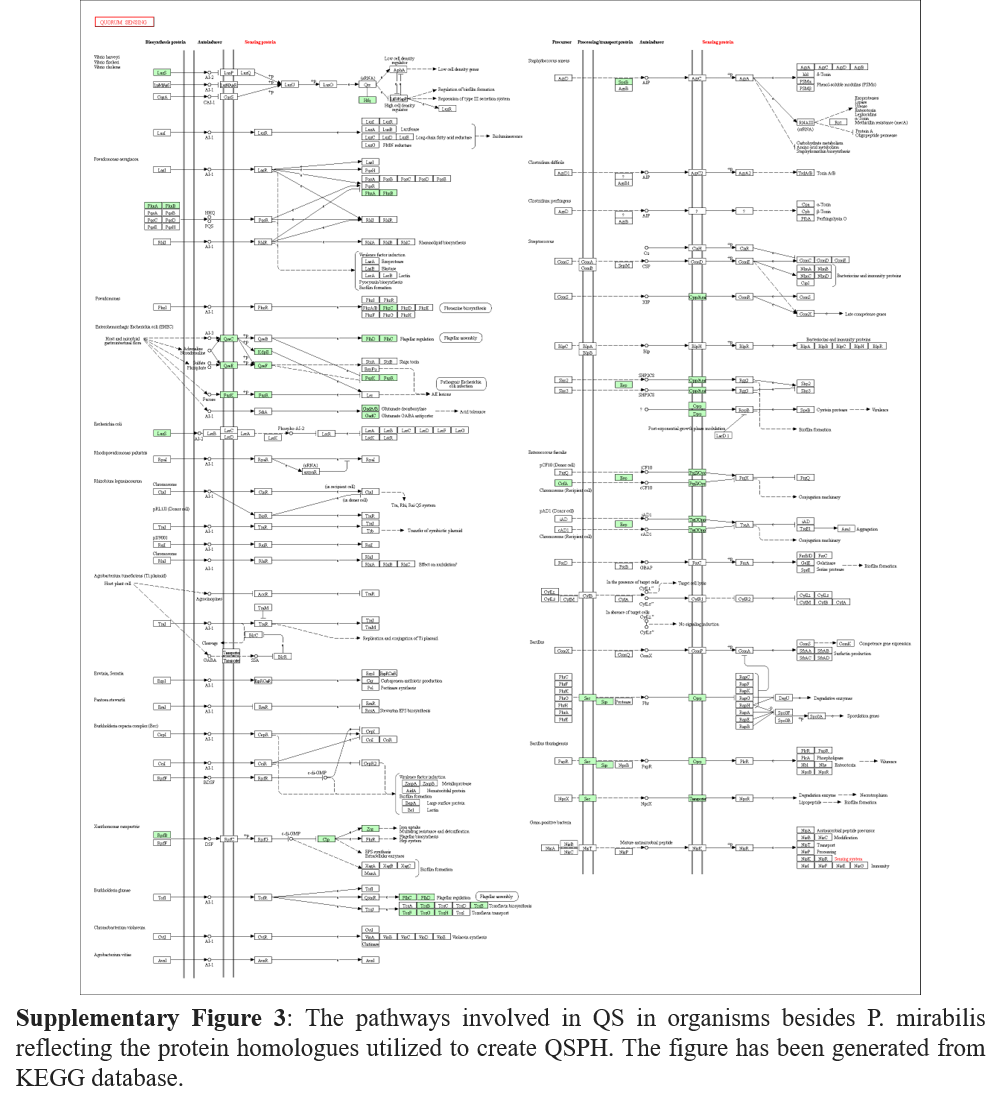

Supplement: Supplementary file 8 [file Image_3.PNG]
